# The Effect of Estradiol Administration on Muscle Mass Loss and Cachexia Progression in Female *Apc*^*Min*/+^ Mice

**DOI:** 10.3389/fendo.2019.00720

**Published:** 2019-11-01

**Authors:** Brittany R. Counts, Dennis K. Fix, Kimbell L. Hetzler, James A. Carson

**Affiliations:** ^1^Integrative Muscle Biology Laboratory, Division of Rehabilitation Sciences, College of Health Professions, University of Tennessee Health Science Center, Memphis, TN, United States; ^2^Department of Exercise Science, University of South Carolina, Columbia, SC, United States

**Keywords:** muscle wasting, cachexia, 17ß-estradiol, hypogonadism, physical activity

## Abstract

Cancer cachexia is a multifactorial muscle wasting condition characterized by severe body weight and muscle mass loss which is secondary to chronic disease. The mechanistic examination of cachexia has predominately focused on the male phenotype and created significant gaps in understanding cachexia progression in the female. Female hypogonadism can accompany cancer cachexia and is characterized by reduced circulating 17ß-estradiol and uterine atrophy. Estrogen has known functions in skeletal muscle homeostasis involving the regulation of muscle protein turnover, cellular stressors, and oxidative metabolism. However, 17ß-estradiol's ability to regulate cachexia progression in the female is not known. The purpose of this study was to determine the effect of gonadal function and estradiol administration on muscle mass loss and cachexia progression in female *Apc*^*Min*/+^ mice.

**Methods:** Female C57BL/6 (B6; *N* = 82) and *Apc*^*Min*/+^ (MIN; *N* = 88) mice were used in two separate experiments. In experiment 1, mice were sacrificed at either 12 (*N* = 20) or 20 (*N* = 41) weeks of age. Body weight and estrous cycle presence was determined weekly. In experiment 2, B6 and MIN mice were randomly allocated to: Control (*N* = 17), received E2 pellet (E2, *N* = 18), ovariectomy surgery (OVX; *N* = 19) or ovariectomy surgery with E2 pellet (OVX + E2; *N* = 21). 17ß-estradiol was administered through an implanted slow-releasing pellet (0.1 mg). In estrogen and ovariectomy experiments, food intake, and functional outcomes were recorded 1 week prior to sacrifice.

**Results:** We report that E2 administration prevented body weight loss, muscle mass loss, cage inactivity, and grip strength loss associated with cachexia. In skeletal muscle, E2 reduced skeletal muscle AMPK phosphorylation, improved mTORC1 signaling, and prevented mitochondrial dysfunction.

**Conclusion:** Our results demonstrate a role for 17ß-estradiol for the prevention of skeletal muscle mass loss in female tumor bearing mice. Furthermore, 17ß-estradiol prevented cachexia's disruption in skeletal muscle signaling involving AMPK and mTORC1, in addition to improving mitochondrial function in female tumor bearing mice.

## Introduction

Cancer cachexia, which occurs in roughly 50% of all cancer patients, is commonly characterized by severe body weight loss. This condition is also associated with several systemic imbalances including anemia, insulin resistance, gonadal dysfunction, and elevated systemic inflammation secondary to chronic disease (1, 2). Patient age is associated with poor prognosis following cancer diagnosis and treatment (3), and the average age of cachexia diagnosis is 68 years (4). Most females are post-menopausal by 55 years of age (5, 6), suggesting that cancer cachexia progression could be influenced by both age and menopausal status. Sex hormones are physiologically relevant throughout the life span, present in almost all tissues, and are linked to disease progression (7, 8). Sex differences have been reported in pre-clinical cancer cachexia models (9, 10) and in cachectic patients (11–14). While females often present a less severe phenotype than males in both pre-clinical models and human cachexia patients, gonadal function has rarely been accounted for in these studies. Furthermore, the mechanistic understanding of cachexia progression has mainly occurred in the male (15, 16). Ovarian function has been reported to be disrupted prior to the initiation of body weight loss and be associated with muscle inflammatory signaling in tumor bearing mice (10). Available evidence clearly demonstrates that further investigation is warranted to mechanistically examine ovarian function's role in cancer cachexia progression.

Estrogen's physiological role has often been investigated in pre-clinical studies through the loss of function or removal of the ovaries (17). Reduced circulating estrogen can decrease bone mineral density, increase abdominal adiposity, increase inflammatory expression, and reduce voluntary activity (18–21). Circulating estrogens can signal through classical or non-genomic activation (22), and regulate skeletal muscle protein turnover and mitochondrial oxidative capacity, which are widely investigated drivers of cancer-induced muscle wasting (20, 23, 24). Moreover, estrogen can regulate skeletal muscle cellular signaling involving Akt, mTORC1, the ubiquitin proteasome system, and 5'-Adenosine monophosphate activated protein kinase (AMPK) (16). Estrogen receptor signaling is also needed for the maintenance of skeletal muscle contractile properties (25). Estrogen combined with progesterone can improve muscle fatigue and voluntary wheel running distance, which are affected by cancer (21, 26). Estrogen's documented effects on skeletal muscle function and cellular processes that regulate protein turnover and metabolism provide justification for further investigation to determine its utility as a therapeutic target for treating muscle wasting in non-hormone sensitive cancers.

The *Apc*^*Min*/+^ (MIN) mouse is an established pre-clinical cachexia model. MIN mice are heterozygotes for adenomatous polyposis coli (*Apc*) gene mutation that causes spontaneous development of intestinal and colon polyps. MIN mice develop non-metastatic intestinal polyps at ~4 weeks of age, which continues until 12–14 weeks of age. Polyp size can continue to increase after 12 weeks of age and is associated with cachexia progression (27). MIN mice initiate body weight loss between 14 and 16 weeks of age (28). We have reported that male MIN mice are hypogonadal (15), have reduced cage activity (29, 30), altered skeletal muscle protein turnover (31), and chronically elevated AMPK (32). Furthermore, male MIN mice demonstrate an induction of skeletal muscle E3 ligase Atrogin-1 during the initiation of bodyweight loss (28, 31). Female MIN mice demonstrate estrous cycle cessation (acyclicity) that corresponds with reduced cage activity, increased fatigue, and muscle mass loss (10). Acyclicity in MIN mice is associated with decreased uterine mass (10). Therefore, the purpose of this study was to determine the effect of gonadal function and estradiol administration on muscle mass loss and cachexia progression in female *Apc*^*Min*/+^ mice. We hypothesized that estradiol administration would prevent cachexia progression and skeletal muscle mass loss in female MIN mice.

## Methods

### Animals

Female C57BL/6 (B6; *N* = 82) and *Apc*^*Min*/+^ (MIN; *N* = 88) mice were bred at the University of South Carolina Animal Resource Facility. MIN mice were initially purchased from Jackson Laboratory (Bar Harbor, ME, USA). Mice were kept on a 12:12 h light/dark cycle beginning at 7:00 a.m. and were given rodent chow *ad libitum* (Harlan Teklad Rodent Diet, #8604, Harlan, Indianapolis, IN, USA). All experiments were approved by the University of South Carolina Institutional Animal Care and Use Committee.

### Experimental Designs

Experiment 1: To determine if the cachectic phenotype in female MIN mice are presented early or late, we sacrificed B6 and MIN mice at 12 (*N* = 20) or 20 (*N* = 41) weeks of age (Table 1). Mice were weighed weekly and we determined the presence (cycling) or absence (acyclicity) of an estrous cycle. In the large cohort of female MIN mice aged 20 weeks, we categorized these mice by cachexia severity to determine the effect of cachexia progression on gonadal function. Mice were stratified by change in body weight from peak: weight stable (0%), initiated (0 to −5%), moderate (−5 to −10%), or severe (<-10%) (Table 2) (10). Then, to further elucidate the importance of estrous cycle presence, MIN mice were stratified based on the presence or absence of the estrous cycle.

**Table 1:** Animal characteristics in female B6 and MIN mice at 12 and 20 weeks.

**Genotype**	**B6**		**MIN**			***p-value***	
					**Age**	**Genotype**	**Interaction**
Age (weeks)	12	20	12	20	<0.001	0.395	0.803
*N*	11	32	9	41			
Sacrifice BW(g)	19.8 (0.3)	21.4 (0.3)	18.4 (0.2)	19.2 (0.3)	0.008	<0.001	0.372
Peak BW(g)	19.8 (0.3)	21.7 (0.3)	18.4 (0.2)	20.7 (0.2)	<0.001	0.002	0.503
BWΔ from Peak (%)	0.0 (0.0)	−0.5 (0.2)	0.0 (0.0)	−6.3 (1.0)^∧^	0.010	0.027	0.027
Percent Cycling (%)	100	95.1	100	53.7			
Total Polyp Number	0	0	41 (7)	53 (4)	0.333	<0.001	0.333
Total Large Polyps (> 2 mm)	0	0	19 (4)	34 (4)	0.102	<0.001	0.102
Hindlimb Muscle (mg)	212 (4)	230 (3)	196 (3)^@^	181 (5)^@^^*^	0.837	<0.001	0.027
Soleus (mg)	5.9 (0.5)	6.7 (0.3)	5.9 (0.5)	6.3 (0.2)	0.080	0.421	0.467
Gastrocnemius (mg)	91.2 (2.3)	98.0 (1.4)	82.6 (1.6)	75.9 (3.1)	0.967	<0.001	0.073
Tibialis Anterior (mg)	34.7 (0.6)	37.4 (0.8)	32.9 (0.9)	29.3 (0.9)^@^^*^	0.815	<0.001	0.029
EDL (mg)	7.4 (0.6)	7.6 (0.3)	7.3 (0.8)	7.1 (0.3)	0.356	0.897	0.408
Spleen (mg)	93 (8)	78 (2)	159 (20)	415 (18)^∧^	<0.001	<0.001	<0.001
Heart (mg)	103 (4)	104 (3)	90 (1)	107 (2)	0.031	0.212	0.068
Epididymal Fat (mg)	255 (16)	265 (15)	196 (36)	86 (14)^∧^	0.045	<0.001	0.017
Tibia (mm)	16.2 (0.1)	16.6 (0.1)	16.1 (0.1)	16.5 (0.1)	0.001	0.405	0.938

*Data is expressed as mean (standard error of the measurement). Mice were sacrificed around 12 or 20 weeks of age. Hindlimb muscle mass includes the sum of the soleus, gastrocnemius, plantaris, tibialis anterior, EDL, and rectus femoris. g, grams; mm, millimeters; mg, milligrams; %, percent; Δ, change; EDL, Extensor Digitorum Longus. Symbols for Interactions*:

∧ *Different than all groups*,

* *Different than B6 12 week, @ Different than B6 20 weeks. p-values for main effects of Age (12 vs. 20 weeks) and Genotype (B6 vs. MIN), and Interactions are listed to the far right of each variable*.

**Table 2:** Female MIN mice characteristics stratified by cachexia severity (Experiment 1).

**Genotype**	**MIN**				
	**WS**	**Initiated**	**Moderate**	**Severe**	***p*-value**
*N*	12	10	9	10	
Sacrifice BW(g)	20.8 (0.4)	19.6 (0.4)	18.8 (0.7)^+^	17.0 (0.3)^∧^	<0.001
Peak BW (g)	21.3 (0.4)	20.4 (0.3)	20.5 (0.4)	20.5 (0.4)	0.341
BWΔ from Peak (%)	−0.1 (0.1)	−2.4 (0.4)	−7.5 (0.7)^+^	−15.3 (1.8)^+^^**^	<0.001
Percent Cycling (%)	100	80	22	0	
Total Polyp	46 (8)	48 (8)	66 (8)	68 (10)	0.141
Total Large Polyps (>2 mm)	32 (7)	31 (9)	39 (9)	36 (8)	0.826
Hindlimb Muscle (mg)	215 (7)	198 (7)	170 (12)^+^	137 (8)^∧^	<0.001
Soleus (mg)	6.4 (0;5)	6.6 (0.4)	6.9 (0.7)	5.6 (0.4)	0.125
Gastrocnemius (mg)	90.8 (3.2)	85.4 (2.6)	69.9 (6.4)^+^	54.5 (3.7)^+^^**^	<0.001
Tibialis Anterior (mg)	34.8 (1.9)	31.1 (1.9)	27.3 (1.9)^+^	23.8 (1.2)^+^^**^	<0.001
EDL (mg)	8.5 (0.5)	7.2 (0.5)	6.4 (0.9)	5.3 (0.5)^+^	0.005
Ovaries (mg)	12 (1)	12 (1)	13 (2)	9 (7)	0.462
Uterus (mg)	43 (4)	36 (4)	33 (5)	38 (3)	0.381
Spleen (mg)	388 (39)	383 (43)	428 (57)	432 (30)	0.357
Heart (mg)	109 (4)	99 (4)	106 (85)	108 (7)	0.501
Epididymal Fat (mg)	156 (29)	118 (25)	51 (51)^+^	5 (5)^∧^	<0.001
Tibia (mm)	16.7 (0.1)	16.4 (0.2)	16.6 (0.1)	16.4 (0.1)	0.108

*Data is expressed as mean (standard error of the measurement). Female MIN mice were sacrificed at ~20 weeks of age and stratified by body weight change from peak. Weight stable (0%), initiated (0 to −5%), moderate (−5 to −10%), and severe (< −10%). Hindlimb muscle mass includes the sum of the soleus, gastrocnemius, plantaris, tibialis anterior, EDL, and rectus femoris. g, grams; mm, millimeters; mg, milligrams; %, percent; Δ, change; EDL, Extensor Digitorum Longus; and WS, Weight Stable. Symbols for Multiple Comparisons*:

∧ *Different than all groups, + Different than WS*,

** *Different than Initiated. P-values are listed to far right for each variable*.

Experiment 2: To determine the effect of 17ß-estradiol administration and ovariectomy surgery on cachexia progression (**Figure 2A**). At 8 weeks of age, B6 and MIN mice were randomly allocated to either; Control (*N* = 17), 17ß-estradiol pellet (E2; *N* = 18), underwent ovariectomy surgery (OVX; *N* = 19) or ovariectomy surgery and received an 17ß-estradiol pellet (OVX + E2; *N* = 21). At 11 weeks of age mice were anesthetized under isoflurane for 5 min for E2 pellet implantation (E2), 30 min to undergo ovariectomy surgery (OVX), E2 pellet implantation and ovariectomy surgery (OVX+E2), or mice were anesthetized under isoflurane for 30 min to receive a SHAM OVX surgery (Intact). A 60-day slow releasing 0.1 mg/pellet of 17ß-estradiol was purchased from Innovative Research of America and used for estrogen administration. Protein expression and mitochondrial respiration was analyzed in B6 and MIN mice that received E2 pellet (E2) or were anesthetized under isoflurane but did not receive a pellet (control). One week prior to sacrifice, grip strength, cage activity, and food intake were recorded in estrogen treated and control mice. At 18 weeks of age, mice were sacrificed following a 5 h fast.

### Cycle Presence

At 10 weeks of age, female B6 and MIN mice were tracked weekly for the presence or absence of an estrous cycle until mice were euthanized (Experiment 1). Herein we have used a modified methodology to limit pseudopregnancy caused by pipette tip insertion (10). Briefly, the mouse was grasped by the base of the tail, and following urination roughly 25–50 μl of PBS was aspirated into the vaginal canal without inserting the pipette tip to avoid pseudopregnancy as previously described (33). Cycle presence was determined by vaginal smears. We examined the presence of squamous epithelial cells. If we observed the absence of an estrous cycle, we continued the vaginal lavage every other day for 1 week to verify a cycle. If a mouse was in diestrus, presented by mostly the presence of leukocytes, vaginal lavages were completed every other day until we observed squamous epithelial cells. Vaginal lavages were completed at least once a week from 10 to 20 weeks of age. Cyclic mice were given a score of 1 and acyclic mice were given a score of 0; allowing us to quantify an estrous cycle index.

### Tissue Collection

Following a 5 h fast, mice were euthanized with a subcutaneous injection of a ketamine-xylazine-acepromazine cocktail (1.4 ml/kg body weight) (34). Hindlimb muscles (soleus, plantaris, extensor digitorum longus, gastrocnemius, tibialis anterior, and rectus femoris) and organs were rapidly excised, cleared of excess connective tissue, rinsed in PBS, weighed, and snap frozen in liquid nitrogen to be analyzed at a later date.

### Intestinal Polyp Quantification

Intestinal segments were excised, cleaned with PBS, cut into equal segments, and stored in 10% neutral formalin until tumor count analysis. Intestinal polyps were analyzed after a deionized water rinse and 0.1% methylene blue staining. Total polyp counts were performed using dissecting micro-scope (model SMZ168, Motic, Xiamen, China) by an investigator blinded to the treatment groups as previously described (28, 35).

### Western Blotting

Western blot analysis was performed as previously described (36). Briefly, frozen gastrocnemius muscle was homogenized in lysis buffer and protein concentration was determined by the Bradford method. Crude gastrocnemius muscle homogenates were fractionated on 10% SDS-polyacrylamide gels and transferred to PVDF membranes. Membranes were stained with Ponceau red to verify equal loading and transfer. Membranes were then blocked at room temperature for 1–2 h in 5% non-fat milk Tris-buffered saline with 0.1% Tween-20 (TBST). Commercially available phosphorylated and total protein primary antibodies for rpS6, 4E-BP1, and AMPK were raised in rabbit. RpS6, 4E-BP1, and AMPK phosphorylation antibodies were expressed relative to total protein on the same gel and quantified as phosphorylation to total ratio. Phosphorylated rpS6 (S240/244) (cat#2215, 1:1000), total rpS6 (cat#2708, 1:2000), phosphorylated 4E-BP1 (T37/44) (cat#2855, 1:1000), total 4EBP1 (cat#9452, 1:2000), phosphorylated AMPK (T172) (cat#, 1:2000), and total AMPK (cat#2603, 1:1000) primary antibodies were purchased from cell signaling. Commercially available total protein primary antibodies for MuRF-1 and Atrogin-1 were raised in rabbit. MuRF-1 and Atrogin-1 proteins were corrected for GAPDH protein expression on the same gel and quantified as total protein expression to GAPDH ratio. Atrogin-1 (cat# AP2041, 1:1000) and MuRF-1 (MP3401; 1:2000) primary antibodies were purchased from ECM biosciences. GAPDH was purchased from cell signaling (cat# 14C10, 1:4000). All primary antibodies were incubated overnight in 5% TBST milk. Membranes were then incubated in 5% milk-TBST containing anti-rabbit (cat#7074, 1:4000) IgG horseradish-peroxidase conjugated secondary antibody purchased from cell signaling for 1 h at room temperature. Enhanced chemiluminescence (ECL) (GE Healthcare Life Sciences, Piscataway, NJ) was used to visualize the antibody-antigen interactions. Membranes were stripped and re-probed for total or GAPDH protein expression. Immunoblot images were collected using a digital imager (SynGene GBox, Frederick, MD) and quantified by densitometry using imaging software (Image J; NIH).

### Mitochondrial Respiration

A randomly selected cohort of five to six mice from control and 17ß-estradiol groups were used for analysis of mitochondrial function. Mitochondrial respiration was measured polarographically in a respiration chamber (Hansatech Instruments, Oxygraph) maintained at 37°C as described previously (37, 38). A 7–10 mg piece of tibialis anterior (TA) muscle was mechanically tweezed with forceps under a dissecting microscope in ice-cold buffer X (mM: 60 K-Mes, 35 KCl, 7.23 K_2_EGTA, 2.77 CaK_2_EGTA, 20 imidazole, 0.5 DTT, 20 taurine, 5.7 ATP, 15 phosphocreatine and 6.56 MgCl_2_, pH 7.1). The fiber bundle was then incubated in 50 μM saponin for 30 min and washed three times for 5 min in respiration buffer (mM: 105 K-Mes, 3 KCl, 1 EGTA, 10 K_2_HPO_4_, 5 MgCl_2_, 0.005 glutamate, 0.002 malate, 0.05% BSA, and 20 creatine, pH 7.1). Fiber bundles were then placed into the oxygraph machine in 20 mM creatine respiration buffer at 37°C and provided with 5 mM of pyruvate and 2 mM of malate to measure complex I-mediated mitochondrial respiration (39, 40). Two minutes after pyruvate and malate, 0.25 mM of ADP was injected into the chamber to induce state three respiration for 5 min. Oligomycin (10 μg ml^−1^) was then injected to induce steady state four respiration for 10 min. The respiratory control ratio (RCR) was calculated by dividing state three by state four respirations. All samples were normalized to dry weight.

### Plasma Estrogen and Progesterone

Immediately prior to sacrifice, blood was collected via retro-orbital sinus with heparinized capillary tubes, placed on ice, and centrifuged (10,000 × g for 10 min at 4°C). The supernatant was removed and plasma 17ß-estradiol (range, 3–300 pg/ml) and progesterone (range, 0.15–40 ng/ml) concentrations were determined by the University of Virginia Center for Research in Reproduction Ligand Assay and Analysis Core Facility.

### Statistical Analysis

All results are reported as means ± standard error of measurement (SEM). To compare sacrifice characteristics of 12 and 20 week B6 and MIN mice, 2 (Age; 12 vs. 20 weeks) × 2 (Genotype; B6 vs. MIN) ANOVA's were used. To compare body weight over time in 20 week B6 and MIN mice, 2 (Genotype; B6 vs. MIN) × 9 (12–20 weeks) repeated measures ANOVA was used. To compare sacrifice characteristics, food intake, and grip strength in MIN mice that had E2 treatment and/or ovariectomy surgery, 2 (Treatment; Control or E2) × 2 (Condition; Intact or OVX) ANOVA's were used. To compare sacrifice characteristics, food intake, and grip strength in B6 mice that had E2 treatment and/or ovariectomy surgery, 2 (Treatment; Control or E2) × 2 (Condition; Intact or OVX) ANOVA's were used. To compare skeletal muscle signaling, mitochondrial respiration, and cage activity in E2 treated B6 and MIN mice, 2 (Genotype; B6 or MIN) × 2 (Treatment; Control or E2) ANOVA's were used. If there was a significant interaction, unpaired student's *t*-test were used to find differences between variables. One-way ANOVA's were used to compare MIN mice sacrifice variables stratified by cachexia severity. Tukey's *post hoc* analysis was used when appropriate. Unpaired Student's *t*-test were used to compare cycling and acyclic MIN mice. Statistical analysis was performed using GraphPad (Prism 8 for MAC OS X, La Jolla, Ca). Level of significance for all measures was set at *p* ≤ 0.05.

### Study Approval

All experiments were approved by the University of South Carolina's Institutional Animal Care and Use Committee.

## Results

### Cachexia in Female Mice (Experiment 1)

Since cachexia progression in the female mouse has not been characterized as well as the male, we first compared B6 and MIN mice at 12 and 20 weeks of age to determine the phenotypic characteristics prior to and during the progression of cachexia (Table 1).

#### Body Weight

**Figure 1:**
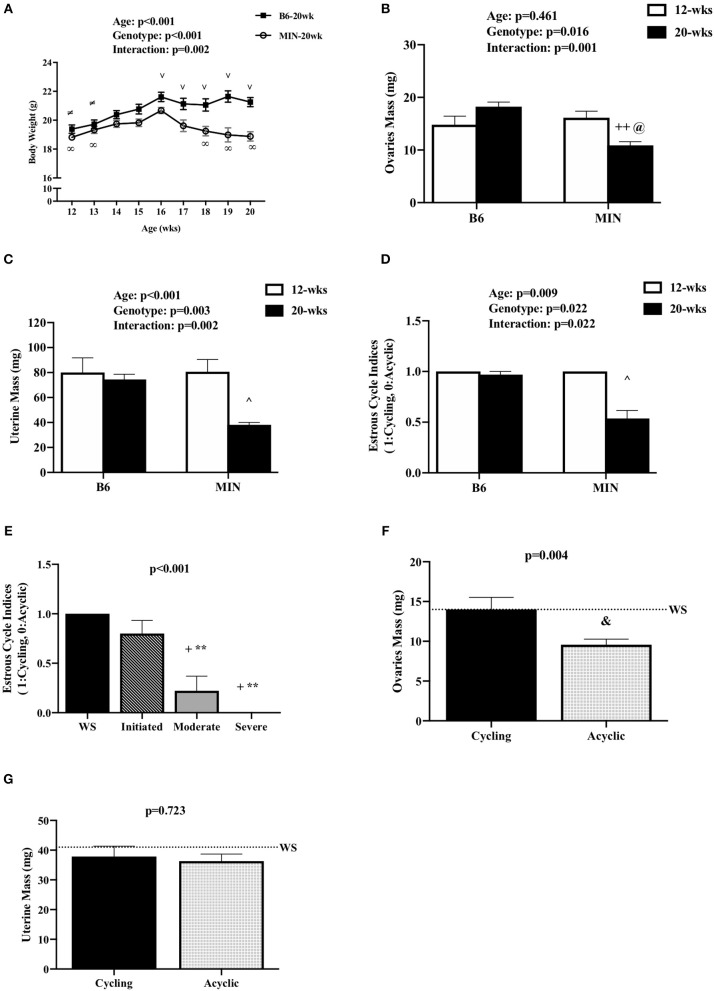
Effect of Cachexia Progression on Hypogonadism. Data is expressed as mean (standard error of the measurement). **(A)** Body weight from 12 to 20 weeks of age in female MIN mice. **(B)** Ovaries mass in 12-week and 20-week B6 and MIN mice. **(C)** Uterine mass in 12-week and 20-week B6 and MIN mice. **(D)** Estrous cycle index in 12-week and 20-week of age. **(E)** Estrous cycle indices in female MIN mice sacrificed at 20 weeks of age stratified by severity. **(F)** Ovaries mass in cycling and acyclic MIN mice. **(G)** Uterine mass in cycling and acyclic MIN mice. Dashed line is a reference point for female Weight Stable (WS) MIN mice. WS, weight stable; mg, milligrams; %, percent; mm, millimeters. Symbols for statistical significance: ∞Different than MIN at 16 weeks, ≠Different than B6 at 16 weeks, and ∨ Different than MIN at given time point, ++Different than 12 week MIN, @Different than 20 week B6, ^∧^Different than all groups, +Different than WS, ^**^Different than Initiated, ^&^Different than Cycling.

We examined body weight change over time in the 20-week-old female mice (Figure 1A). MIN mice at 20 weeks of age demonstrated body weight loss which was decreased in the MIN between 16 and 18 weeks of age. Additionally, B6 body weight at 16 weeks of age was greater than the MINs at this age. MIN mice did exhibit a change in body weight at 20 weeks of age when compared to all groups (Table 1). Regardless of genotype, mice grew after 12 weeks of age. Body weight at 20 weeks of age was increased when compared to 12-week-old mice (Table 1). However, there was a main effect of genotype for MIN peak bodyweight to be less than B6 peak bodyweight.

#### Skeletal Muscle and Fat Mass

Total hindlimb muscle mass in 20-week-old MIN mice was decreased when compared to 20-week-old B6 mice (Table 1). We also examined individual hindlimb muscles to examine the effect of muscle phenotype and wasting. Regardless of age, MIN gastrocnemius muscle mass was decreased in MIN mice compared to B6 mice. MIN tibialis anterior muscle mass at 20 weeks of age was reduced when compared to 20-week-old B6 mice. Interestingly, neither the soleus nor extensor digitorum longus muscles demonstrated any differences between treatment groups. Epididymal fat mass in the 20-week-old MIN mice was decreased compared to all other treatment groups.

#### Other Cachexia Related Variables

Total intestinal and colon polyp number and the large size polyp number were not different between 12 and 20-week-old MIN mice (Table 1). Spleen mass in the 20-week-old MIN mice was increased when compared to all other treatment groups (Table 1).

### Characterization of Cachexia Progression (Experiment 1)

Cachexia progression is often characterized by the body weight change from the peak body weight measured during the study. However, we have previously reported that female MIN mice exhibit more variable body weight change at any given age when compared to male MIN mice. Therefore, we stratified 20-week-old MIN mice by the degree of body weight change and examined phenotype characteristics. Twenty-week female MIN mice were stratified by their body weight change from peak body weight (Table 2). Mice were classified as weight stable, initiating cachexia, moderate cachexia, or severe cachexia (10). As expected, body weight change in this cohort of mice exhibited significant differences in the cachexia severity classification. There was a difference in body weight change between the weight stable and moderate cachexia groups. The body weight change in the severe cachexia group was larger than the change in the weight stable and initiating cachexia groups. There were no differences between groups for peak body weight and tibia length, suggesting that body size was not altered by cachexia. Interestingly, there were no differences in either total polyp number or large polyp number between groups (Table 2). Hindlimb muscle mass in the moderate cachexia group was decreased compared to weight stable mice, and mice with severe cachexia had less hindlimb muscle mass when compared to all other groups. Hindlimb muscles were also analyzed individually, and exhibited differences related to mass loss during the progression of cachexia. Both gastrocnemius and tibialis anterior muscle mass were decreased in the moderate cachexia group when compared to weight stable mice. In the severe cachexia group gastrocnemius muscle mass and tibialis anterior muscle mass were reduced compared to both weight stable and initiating cachexia groups. Extensor digitorum longus muscle mass was decreased with severe cachexia when compared to weight stable mice. Interestingly, soleus muscle mass was not altered by the cachexia classification. These results suggest that skeletal muscle mass loss in the cachectic female occurs after the initiation of cachexia, and muscle phenotype impacts the degree of mass loss. Epididymal fat mass was decreased by moderate cachexia when compared to weight stable mice, and severely cachectic mice had less epididymal fat mass than all other cachexia classifications. Taken together, our results suggest that there is a clear phenotypic distinction that occurs during the progression of cachexia in the female MIN mouse. While fat loss continued to decline with greater body weight change, muscle mass loss occurred prior to severe weight loss. Lastly, the initiation of cachexia provides a critical window for intervening to slow muscle and fat mass loss in the female.

### Hypogonadism and Cachexia Progression (Experiment 1)

We examined hypogonadism during the progression of cachexia. Gonadal mass and estrous cycle index were examined in 12 and 20-week-old mice. The 20-week old MIN mice exhibited hypogonadism. Ovary mass was decreased in 20-week-old MIN mice when compared to the same age B6 mice and 12-week-old MIN mice (Figure 1B). Uterine mass in the 20-week-old MIN mice was decreased compared to all other groups (Figure 1C). The estrous cycle index was also decreased in the 20-week-old MIN when compared to all other groups (Figure 1D). To determine if hypogonadism preceded cachexia development, we examined gonadal function in 20 week female MIN mice stratified by cachexia severity (Table 2). Neither ovary nor uterine mass were impacted by cachexia severity, demonstrating that hypogonadism occurs before bodyweight loss. The estrous cycle index was reduced in moderate and severely cachectic mice when compared to weight stable and mice initiating cachexia (Figure 1E). These data suggest that the estrous cycle is maintained until moderate body weight loss occurs.

Given that estrous cycle absence is a possible indicator of cachexia progression, we examined the phenotypic differences in the presence or absence of an estrous cycle. Mice were stratified by cyclicity (*N* = 22) or acyclicity (*N* = 19). We found that the change in body weight from peak was greater in acyclic mice when compared to cyclic mice [cyclic: −1.2 (0.38%), acyclic: −11.4 (1.4%); *data not shown*]. Hindlimb muscle mass [cyclic: 209 (5 mg), acyclic: 150 (7 mg); *data not shown*] and epididymal fat mass [cyclic: 145 (23 mg), acyclic: 26 (15 mg); *data not shown*] were also decreased in acyclic mice when compared to cyclic mice. Ovary mass decreased in acyclic compared to cyclic mice (Figure 1F). Total polyp number (data not shown) and uterine mass (Figure 1G) were not affected by estrous cyclicity, which is in agreement with previous published data that rigorously examined the difference in acyclic and cyclic female MIN mice (10). Collectively, our data suggest that estrous cycle cessation occurs after a mouse has achieved 5% body weight loss, and acyclic mice exhibit a more severe cachectic phenotype.

### Ovariectomy and 17ß-estradiol Administration and Cachexia Progression (Experiment 2)

**Figure 2:**
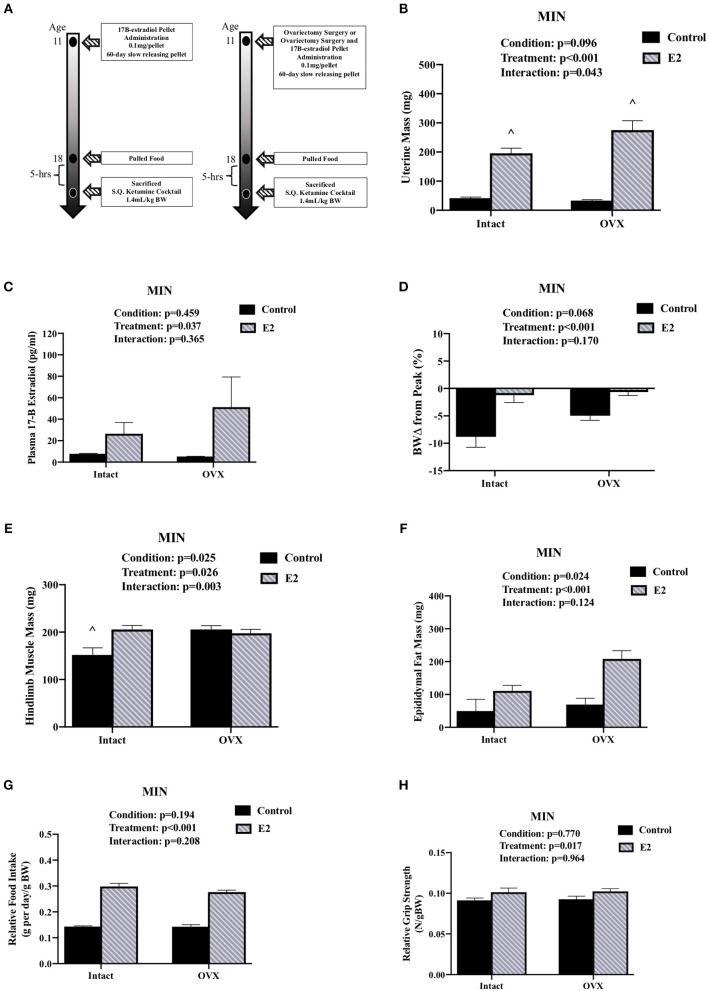
Ovariectomy and 17ß-Estradiol Administration on Cachexia in Female MIN Mice. Data is expressed as mean (standard error of the measurement). All female mice analyzed correspond to Table 3. **(A)** Study Design. At 11 weeks of age female MIN mice were randomly allocated to intact control, 17ß-Estradiol pellet (E2), ovariectomy surgery (OVX), or ovariectomy surgery followed by 17ß-estradiol pellet (OVX + E2). Intact mice underwent a SHAM OVX surgery. **(B)** Uterine mass. **(C)** Plasma concentration of 17ß-Estradiol. **(D)** Body weight change from peak. **(E)** Hindlimb muscle mass. **(F)** Epididymal fat mass. **(G)** Relative food intake. **(H)** Relative grip strength. E2, 17ß-estradiol; mg, milligrams; pg/ml, picograms per milliliter; g, grams. Food intake was collected on a subset of mice (*N* = 5–7 per group). *p*-values for main effects of Condition (intact vs. OVX) and Treatment (Control vs. E2), and Interactions are listed above the figures. Symbol for an Interaction: ^∧^Different than all groups.

We investigated the importance of gonadal hormones in the regulation of cachexia progression. The effect of ovariectomy and 17ß-estradiol administration on cachexia progression in female MIN mice was examined (Table 3). At 8 weeks of age, mice were randomly to either; SHAM OVX surgery (intact control), 17ß-estradiol pellet administration (intact E2), ovariectomy (OVX control), or ovariectomy and 17ß-estradiol pellet administration (OVX+E2). At 11 weeks of age, mice underwent surgery and/or pellet implantation and were sacrificed at 18 weeks of age (Figure 2A). Uterine mass was increased in ovary intact MIN mice receiving E2 when compared to intact control and OVX control. Uterine mass was increased in OVX mice that received E2 compared to all other groups (Figure 2B). There was a main effect of treatment for E2 mice to increase circulating 17ß-estradiol compared to control mice (Figure 2C). There were main effects of treatment for E2 to have reduced MIN mouse body weight change (Figure 2D), increased epididymal fat mass (Figure 2F), increased relative food intake (Figure 2G), increased relative grip strength (Figure 2H), and increased liver mass (Table 3) compared to control MIN mice. There were main effects of condition for OVX to increase circulating progesterone (Table 3) and increase epididymal fat mass (Figure 2F) compared to intact MIN mice. While there were no differences in pre-treatment body weights (Table 3), OVX mid-treatment (14 weeks) body weight was increased when compared to all groups. Hindlimb muscle mass was decreased in the intact control MIN mice when compared to all other groups (Figure 2E). There were no differences between treatment groups in total polyp number, large polyp number, spleen mass, and heart mass. Taken together, 17ß-estradiol was able to prevent body weight loss in the MIN mouse and improve indices of cachexia without altering total polyp burden.

**Table 3:** Ovariectomy and 17ß-estradiol treatment characteristics of female MIN mice (Experiment 2).

**Genotype**	**MIN**				
	**MIN Intact**		**MIN OVX**		***p-value***		
**Treatment**	**Control**	***E2***	**OVX**	**OVX ±*E2***	
					**Condition**	**Treatment**	**Interaction**
*N*	8	9	10	11			
BW Pre-Treatment (g)	18.4 (0.5)	17.9 (0.4)	18.8 (0.3)	18.6 (0.4)	0.216	0.482	0.757
BW Mid-Treatment (g)	18.6 (0.4)	20.3 (0.4)	22.7 (0.5)^∧^	20.1 (0.4)	<0.001	0.383	<0.001
BW End-Treatment (g)	18.7 (0.5)^∧^	21.4 (0.5)	21.3 (0.2)	21.0 (0.5)	0.021	0.009	0.002
Peak BW (g)	20.4 (0.2)	21.7 (0.4)	22.7 (0.3)^$$^	21.3 (0.5)	0.054	0.873	0.003
Total Polyp Number	77 (13)	57 (7)	53 (14)	52 (18)	0.288	0.440	0.491
Total Large Polyps (> 2 mm)	53 (10)	29 (6)	30 (3)	39 (5)	0.500	0.407	0.081
Progesterone (ng/ml)	1.8 (0.2)	2.3 (0.6)	5.2 (0.7)	4.3 (1.2)	0.001	0.796	0.392
Liver (mg)	1150 (72)	1606 (100)	1281 (44)	1552 (97)	0.644	<0.001	0.268
Spleen (mg)	422 (47)	520 (52)	342 (37)	400 (57)	0.052	0.124	0.691
Heart (mg)	100 (7)	116 (10)	115 (3)	108 (4)	0.591	0.454	0.069
Tibia (mm)	16.5 (0.1)	16.2 (0.1)	16.3 (0.1)	15.9 (0.1)	0.061	0.017	0.982

*Data is expressed as mean (standard error of the measurement). Female MIN mice were sacrificed at 18 weeks of age following a 5-h fast. MIN mice were randomly assigned to intact control, received 17ß-Estradiol pellet (intact E2), underwent ovariectomy surgery (OVX) or ovariectomy surgery and received a 17ß-Estradiol pellet (OVX + E2). Intact mice underwent a SHAM OVX surgery. g, grams; mm, millimeters; mg, milligrams; %, percent; ng, nanograms; E2, 17ß-Estradiol; OVX, Ovariectomy. Symbols for Interactions*:

∧ *Different than all groups, $$ Different than intact control. P-values for main effects of Condition (intact vs. OVX) and Treatment (Control vs. E2), and Interactions are listed to the far right of each variable*.

We also examined the effect of ovariectomy and 17ß-estradiol administration in female B6 mice (Table 4). There were main effects of treatment for E2 mice to have increased uterine mass, increased circulating 17ß-estradiol, and decreased relative grip strength compared to control mice (Table 4). There was also a main effect of condition for OVX in B6 mice to decrease uterine mass, increase circulating progesterone, and decrease relative grip strength compared to intact B6 mice (Table 4). Intact E2 mice had a greater body weight at the pre, mid, and end time points. Peak body weight was also greater in the intact E2 B6 mice when compared to intact control B6 mice. Hindlimb muscle mass was not altered by either OVX or E2 treatments in B6 mice. Interestingly, epididymal fat mass was decreased in OVX+E2 B6 mice when compared to either intact or OVX control mice. Epididymal fat mass was also decreased in intact E2 B6 mice when compared to intact control mice. Relative food intake was reduced in the intact control pellet mice when compared to all other treatment groups. Intact E2 mice had reduced relative food intake compared to either B6 OVX and OVX + E2 mice. Taken together, these results validate our 17ß-estradiol administration and OVX treatments and identify differences when examining the effects of ovariectomy or E2 administration in either healthy or tumor bearing mice.

**Table 4:** Ovariectomy and 17ß-estradiol treatment characteristics of female B6 mice (Experiment 2).

**Genotype**	**B6**				
	**B6 Intact**		**B6 OVX**				
**Treatment**	**Control**	***E2***	**OVX**	**OVX ±*E2***		***p-value***	
					**Condition**	**Treatment**	**Interaction**
*N*	9	11	9	10			
BW Pre-Treatment (g)	17.5 (0.2)	18.5 (0.3)^$$^	18.1 (0.3)	17.7 (0.3)	0.687	0.330	0.010
BW Mid-Treatment (g)	19.1 (0.3)	20.0 (0.3)^$$^	20.9 (0.3)	20.2 (0.3)	0.004	0.952	0.026
BW End-Treatment (g)	20.4 (0.2)	21.5 (0.5)^$$^	22.2 (0.4)	21.6 (0.3)	0.021	0.584	0.037
Peak BW (g)	20.5 (0.2)	21.5 (0.5)^$$^	22.5 (0.4)	21.7 (0.3)	0.009	0.788	0.040
Uterus Mass (mg)	81 (6)	238 (11)	39 (4)	203 (17)	0.001	<0.001	0.770
Hindlimb Muscle Mass (mg)	218 (5)	220 (4)	225 (6)	220 (5)	0.457	0.790	0.461
Epididymal Fat (mg)	324 (24)	166 (33)^$$^	349 (21)	72 (24)^$$^^*^	0.233	<0.001	0.045
Liver (mg)	894 (33)	1011 (26)	1173 (42)^∧^	1015 (27)	<0.001	0.550	<0.001
Heart (mg)	86 (1)	99 (13)	96 (3)	104 (4)	0.035	0.005	0.431
Tibia (mm)	16.4 (0.1)	16.5 (0.1)	16.7 (0.1)	16.4 (0.1) ^*^	0.302	0.142	0.010
Relative Food Intake (g/g BW)	0.14 (0.00)^∧^	0.19 (0.01)^∧^	0.20 (0.01)	0.20 (0.01)	<0.001	<0.001	<0.001
Relative Grip Strength (N/g BW)	0.11 (0.00)	0.10 (0.00)	0.09 (0.00)	0.08 (0.00)	<0.001	0.018	0.911
Plasma 17-B Estradiol (pg/ml)	5.3 (0.3)	163 (41)	3.0 (0.8)	99 (43)	0.298	<0.001	0.334
Plasma Progesterone (ng/ml)	3.6 (1.0)	4.5 (0.8)	6.6 (1.3)	5.5 (1.1)	0.048	0.933	0.217

*Data is expressed as mean (standard error of the measurement). Female B6 mice were sacrificed at 18 weeks of age following a 5-h fast. B6 mice were randomly assigned to intact control, received 17ß-Estradiol pellet (intact E2), underwent ovariectomy surgery (OVX) or ovariectomy surgery and received a 17ß-Estradiol pellet (OVX + E2). Intact mice underwent a SHAM OVX surgery. g, grams; mm, millimeters; mg, milligrams; %, percent; ng, nanograms; E2, 17ß-Estradiol; OVX, Ovariectomy. Food intake was collected on a subset of mice (N = 5–7 per group). Symbols for Interactions*:

∧ *Different than all groups, $$ Different than intact control*,

* *Different than OVX. P-values for main effects of Condition (intact vs. OVX) and Treatment (Control vs. E2), and Interactions are listed to the far right for each variable*.

### 17ß-Estradiol Administration and Skeletal Muscle Signaling (Experiment 2)

**Figure 3:**
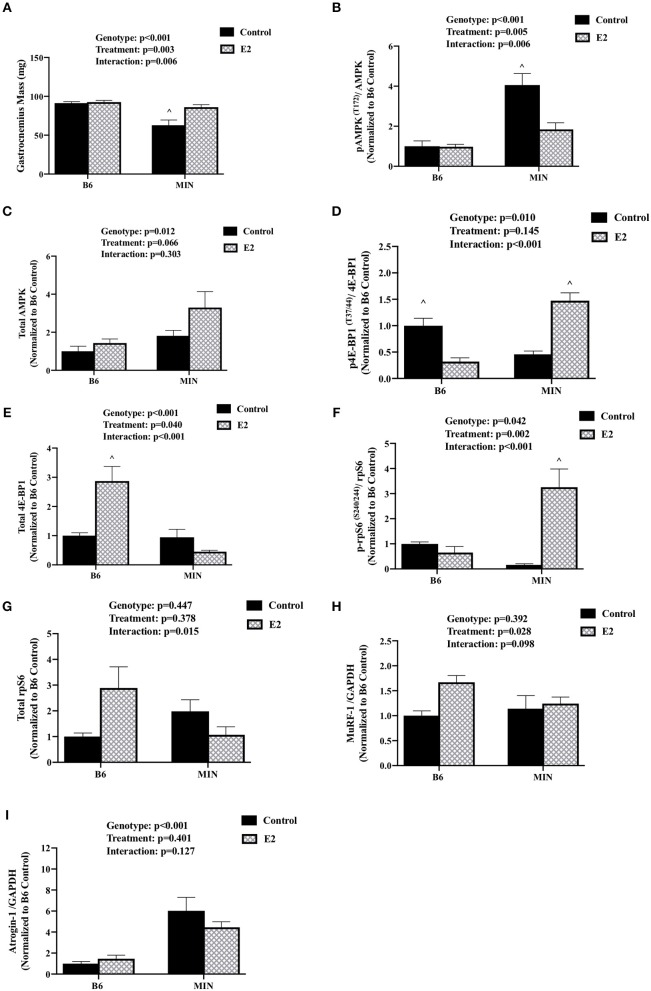
Effect of 17ß-estradiol administration on skeletal muscle signaling. Data is expressed as mean (standard error of the measurement). At 11 weeks of age female MIN mice were randomly allocated to control or 17ß-Estradiol pellet (E2) in B6 and MIN mice. **(A)** Gastrocnemius muscle mass. **(B)** Phosphorylation at site T172 to total ratio of AMPK. **(C)** Total AMPK protein expression. **(D)** Phosphorylation at site T37/44 to total ratio of 4E-BP1. **(E)** 4E-BP1 total protein expression. **(F)** Phosphorylation at site S240/244 to total ratio of rpS6. **(G)** rpS6 total protein expression. **(H)** MuRF-1 to GADPH ratio. **(I)** Atrogin-1 to GAPDH ratio. Data was normalized to B6 control for all protein analysis. E2, 17ß-estradiol; mg, milligrams; AMPK, 5-AMP-activated protein kinase; 4E-BP1, 4E-Binding Protein 1; rpS6, ribosomal protein S6; MuRF-1, Muscle Ring Finger Protein-1; GAPDH, Glyceraldehyde 3-phosphate dehydrogenase. *p*-values for main effects of Genotype (B6 vs. MIN) and Treatment (Control vs. E2), and Interactions are listed above the figures. Symbols for Interaction: ^∧^Different than all groups.

We determined the effect of E2 administration on skeletal muscle signaling associated with cancer cachexia. Skeletal muscle AMPK has been widely investigated for its regulatory role in protein turnover and metabolism. AMPK is chronically activated in cachectic male MIN mouse skeletal muscle and further increased by high levels of circulating IL-6. Skeletal muscle mTORC1 signaling through downstream targets 4E-BP1 and rpS6 is also suppressed by cachexia and IL-6 (32). Protein expression was examined in the gastrocnemius muscle. MIN control mice had decreased gastrocnemius muscle mass when compared to all other groups (Figure 3A). E2 treatment increased MIN gastrocnemius muscle mass to the level of the B6 mouse. In B6 mice, muscle AMPK (T172) phosphorylation to total ratio was not different than B6 mice administered E2 (Figure 3B). As expected, muscle AMPK (T172) phosphorylation to total ratio was increased in MIN control pellet mice compared to both B6 groups; E2 treatment in the MIN mouse suppressed this induction (Figure 3B). There was a main effect of genotype for MIN mice to have increased total muscle AMPK protein expression compared to B6 mice (Figure 3C). 4E-BP1 (T37/44) phosphorylation to total ratio was decreased in B6 E2 treatment mice compared to B6 control mice. The 4E-BP1 (T37/44) phosphorylation to total ratio was decreased in MIN control mice when compared to B6 control mice. E2 treatment in MIN mice increased 4E-BP1 phosphorylation to total ratio compared to MIN control mice (Figure 3D). Interestingly, E2 treatment to B6 mice increased total 4E-BP1 above all other groups (Figure 3E). Muscle rpS6 (S240/244) phosphorylation to total ratio was increased by E2 treatment in MIN mice compared to all other groups (Figure 3F). There were no differences in total rpS6 protein expression (Figure 3G). The protein expression of muscle E3 ligases involved in ubiquitin proteasome degradation were also examined. We report differential sensitivity of MuRF-1 and Atrogin-1 protein expression to either cachexia or E2 stimuli. There was a main effect of treatment for E2 mice to increase muscle MuRF-1 protein expression compared to control mice (Figure 3H). We report a doublet of MuRF-1 which the scientific literature seems to be equivocal on whether MuRF-1 is expressed as a singlet or doublet and as to the importance of the doublet, research is warranted to further define the biological significance of the MuRF-1 doublet. Published studies suggest that the MuRF-1 double band can be associated with the gel percentage used, animal phenotype, or injury recovery (41–45). However, the specific rationale for the cause of the double bands has not been firmly established and warrants further investigation. There was a main effect of genotype for MIN mice to have increased muscle Atrogin-1 protein expression compared to B6 mice (Figure 3I). While we report the ratio of phosphorylated to total protein to represent activity, we also quantified muscle AMPK, rpS6, and 4E-BP1 phosphorylation separately (Supplemental Figure 1). There was a main effect of genotype for MIN mice to have increased muscle AMPK (T172) phosphorylation compared to B6 mice. However, muscle 4E-BP1(T37/44) phosphorylation in MIN control mice was reduced when compared to B6 control mice. MIN E2 treated mice had higher rpS6 (S240/244) phosphorylation when compared to MIN control or B6 E2 treated mice. Taken together, these data suggest that increased plasma 17ß-estradiol can prevent muscle mass loss and improve some aspects of disrupted AMPK and mTORC1 skeletal muscle signaling in female tumor bearing mice.

### 17ß-Estradiol Administration and Muscle Mitochondrial Respiration (Experiment 2)

**Figure 4:**
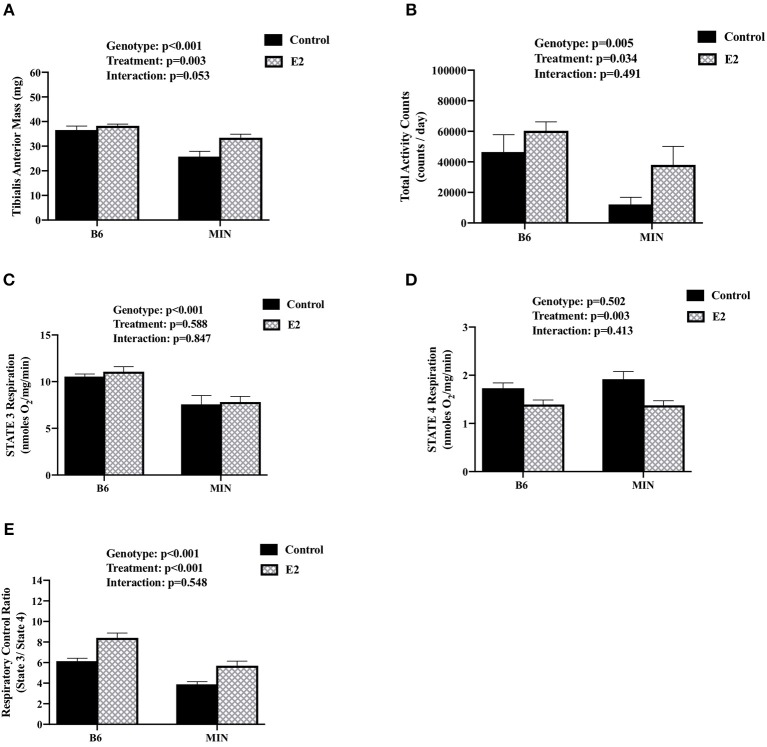
Effect of 17ß-estradiol administration on skeletal muscle mitochondrial respiration. Data is expressed as mean (standard error of the measurement). At 11 weeks of age female MIN mice were randomly allocated to control or 17ß-Estradiol pellet (E2) in B6 and MIN mice. **(A)** Tibialis anterior muscle mass. **(B)** Total ambulatory cage activity counts for 24-h. **(C)** STATE 3 mitochondrial respiration. **(D)** STATE 4 mitochondrial respiration. **(E)** Mitochondrial respiratory control ratio (STATE 3/STATE 4). Physical activity was collected on a subset of mice (*N* = 5–6 per group). Mitochondrial respiration was collected on a subset of mice (*N* = 5–6 per group). E2, 17ß-estradiol; mg, milligrams; counts/day, cage activity counts per day; nmoles/0_2_/mg/min, nanomoles of 0_2_ per milligram of muscle per minutes. P-values for main effects of Genotype (B6 vs. MIN) and Treatment (Control vs. E2); and Interactions are listed above the figures.

We examined the effect of 17ß-estradiol administration on skeletal muscle mitochondrial respiration from isolated tibialis anterior fiber bundles. Estrogen has established beneficial effects on mitochondrial function. Furthermore, mitochondria dysfunction has been widely investigated as a driver of cancer-induced muscle wasting. Tibialis anterior muscle mass was sensitive to both cachexia and E2 treatment (Figure 4A). There was a main effect of genotype for MIN mice to have decreased tibialis anterior muscle mass compared to B6 mice. There was a main effect of treatment for E2 mice to have increased tibialis anterior muscle mass compared to control mice. Mouse cage activity level has been shown to be suppressed during the progression of cancer cachexia, and extended periods of inactivity can impact skeletal muscle oxidative metabolism. Mouse cage activity was impacted by the cancer environment and E2 treatment. There was a main effect of genotype for MIN mice to have decreased cage activity compared to B6 mice and a main effect of E2 treatment of have increased cage activity compared to control mice (Figure 4B). There was a main effect of genotype for MIN mice to have decreased STATE 3 respiration (Figure 4C) and decreased respiratory control ratio (Figure 4E) compared to B6 mice. However, there was a main effect of treatment for E2 mice to have decreased STATE 4 respiration (Figure 4D) and increase the muscle respiratory control ratio (Figure 4E) compared to control mice. Collectively, these data suggest that 17ß-estradiol increased cage activity and prevented mitochondrial respiration dysfunction in female MIN mice.

## Discussion

There is evidence to link hypogonadism and cancer cachexia progression in the MIN mouse (10, 15). There is also sexual dimorphism in the progression of cancer cachexia that has only just begun to be investigated (46). We extended these findings by elucidating the therapeutic potential of estrogen on the progression of cachexia in tumor bearing mice. In female MIN mice, 17ß-estradiol administration prevented muscle mass loss and blocked whole body indices of cachexia progression involving grip strength loss and decreased cage activity. Interestingly, 17ß-estradiol administration increased relative food intake in MIN mice, which was likely involved in the prevention of muscle mass loss. However, food intake has not been reported to be reduced in male and female MIN mice undergoing cachexia. The beneficial effects of 17ß-estradiol administration in ovariectomized MIN mice suggest that ovarian function is not necessary for the beneficial effects on cachexia progression, and has significant therapeutic implications since most woman that develop cachexia are hypogonadal (47).

Ovarian estrous cycle cessation is associated with cachexia progression in female MIN mice and has been reported to precede the development of cachexia (10). We extend these findings, reporting that estrous cycle cessation is more prevalent after the mouse has achieved 5% body weight loss. Interestingly, muscle mass, and epididymal fat loss continue to decline as does body weight, which further emphasizes the importance of gonadal dysfunction in the progression of cancer cachexia. While not affected by cachexia severity, uterine and ovaries mass decreased between 12 and 20 weeks of age in tumor bearing mice, and indices of ovarian cycling were also reduced by 20 weeks of age. Thus, the cancer environment and cachexia differentially affect ovarian function. We have previously shown that the ovaries being present in female tumor bearing mice can protect mice from high levels of circulating IL-6 (10). Interestingly, we report an increase in progesterone in ovariectomized mice. The ovariectomized mice progesterone concentrations were within the normal range as previously reported for ovariectomized female mice (48). Additionally, throughout the estrous cycle progesterone can range from 2 to 8 ng/ml (49) thus the increase in progesterone in ovariectomized mice is most likely negligible. It is possible that estradiol concentrations are different between B6 and MIN mice, but is greatly influenced by the estrous cycle phase which can range from 5 to 60 pg/ml (50), and is most likely a result of the estrous cycle.

Skeletal muscle mass is maintained by the balanced regulation of protein synthesis and degradation (51), and cancer-induced disruption to this regulation is a well-established driver of cachexia (31, 52, 53). We examined the effect of 17ß-estradiol on cachectic skeletal muscle protein turnover. Estrogen can regulate mTORC1 signaling, the ubiquitin proteasome system, autophagy, and AMPK signaling (16). All of these signaling pathways have been reported to be disrupted in cachectic skeletal muscle. We found that 17ß-estradiol inhibited the cachexia induction of AMPK, which coincided with the induction of mTORC1 signaling and suggests a role for 17ß-estradiol being an anabolic stimulus in the catabolic environment. Our results suggest differential regulation of mTORC1 by 17ß-estradiol and the cachexic environment when compared with healthy wild type mice. There was no induction of mTORC1 signaling in wildtype mice receiving 17ß-estradiol, which coincides with reports that postmenopausal woman have no change in protein synthesis following estradiol administration (54, 55). In cultured myotubes estradiol improved protein synthesis further confounding the implications of estradiol in skeletal muscle signaling in a non-diseased state (56). Progesterone has been implicated as an anabolic stimulant (55), whereas estradiol may have greater effects on cellular apoptosis and contractile properties (24). Furthermore, we report that Atrogin-1, not MuRF-1 was induced in MIN mouse skeletal muscle. These results coincide with previous published findings from our lab reporting the induction of muscle Atrogin-1 expression in MIN mice without changes in MuRF-1 expression (28). Additionally, we report MuRF-1 expression was induced by 17ß-estradiol administration. The induction of MuRF-1 following 17ß-estradiol administration is an agreement with previous human studies reporting an induction of MuRF-1, but not Atrogin-1, in post-menopausal women following hormone replacement therapy (57). Further research is warranted to determine if 17ß-estradiol can prevent skeletal muscle mass wasting through direct regulation of AMPK and mTORC1 signaling. Collectively, suggesting a role for sex hormones to minimize the effects of hypogonadism through muscle anabolism.

Skeletal muscle mitochondrial dysfunction has been widely investigated as a critical driver of muscle wasting with cancer and aging (58, 59). Furthermore, mitochondrial dysfunction is associated with AMPK activation in disease and has the potential to disrupt mTORC1 activity in male MIN mice (60). We report that mitochondrial State 3 respiration was decreased in tumor bearing mice whereas state four mitochondrial respiration was decreased in mice that received 17ß-estradiol. These findings suggest that tumor bearing mice have suppressed substrate mitochondrial oxidation and improved proton leak (61). These findings are further supported by increased respiratory control ratio (RCR: state 3/state 4) indicative of mitochondrial function, such that 17ß-estradiol was able to prevent RCR suppression in MIN mice. These results are similar to reported improvements of 17ß-estradiol administration on muscle mitochondrial function in disease free mice (23). Estrogen is an established modulator of muscle mitochondrial biogenesis and mitophagy (62), induces mitochondrial gene expression (23), and improves ATP turnover (63). Reduced physical activity is a common altered behavior in pre-clinical models of cancer cachexia and cachectic patients (30, 64, 65), and decreased activity level can impact mitochondrial function. Improved mitochondrial respiration reported herein coincides with increased activity in the E2 treated MIN mice. 17ß-estradiol administered to MIN mice prevented physical activity loss which accompanies cachexia progression, and it has been reported that in OVX mice, estradiol improved voluntary wheel activity (26), thus having significant clinical implications. Furthermore, cancer cachexia can disrupt normal feeding and physical activity behavior patterns (66, 67) and has been suggested to be a potential driving factor in cancer biology (68). Further research is warranted to determine how estradiol was able to protect skeletal muscle mitochondrial function in the cachectic environment, and if this effect was related to improved feeding and activity behaviors.

In summary, to best of our knowledge this is the first study to examine 17ß-estradiol's effect on cachexia progression in female tumor bearing mice. Our findings support a role for 17ß-estradiol administration in the prevention of cachexia in the female MIN mouse. These findings have important implications for therapeutic treatment options for cachectic patients with non-hormone sensitive cancer. We have shown that 17ß-estradiol administration can prevent skeletal muscle mass loss in female MIN mice with or without the ovaries being present. Furthermore, 17ß-estradiol normalized cachexia's disruption to skeletal muscle AMPK signaling, mTORC1 signaling, and mitochondrial function. Indices of decreased function in cachectic tumor bearing mice involving reduced grip strength and decreased cage activity level were prevented by 17ß-estradiol administration. We also report that the estrous cycle in tumor bearing mice is associated with cachexia progression. Our results suggest a role for hypogonadism in cachexia's progression, and further research is warranted to determine if this is a viable therapeutic target for the management of cachexia with some types of cancers. Further investigation is needed to determine the mechanisms by which 17ß-estradiol can target muscle fiber intracellular signaling and the associated microenvironment in muscle to alter wasting associated with the progression of cachexia. This additional knowledge should provide valuable insight on the regulation of cachexia progression and the development of efficacious treatments for this wasting disease.

## Data Availability Statement

The datasets generated for this study are available on request to the corresponding author.

## Ethics Statement

All experiments were approved by the University of South Carolina's Institutional Animal Care and Use Committee.

## Author Contributions

BC and DF contributed to collecting data. BC, DF, and JC contributed to data interpretation and contributed to writing and preparing the manuscript. KH and JC contributed to the conceptual development the study.

### Conflict of Interest

The authors declare that the research was conducted in the absence of any commercial or financial relationships that could be construed as a potential conflict of interest.
