# Supplementary figures and images for: The Effect of Estradiol Administration on Muscle Mass Loss and Cachexia Progression in Female ApcMin/+ Mice
Source: Front Endocrinol (Lausanne). 2019 Nov 1;10:720. doi: 10.3389/fendo.2019.00720 (PMC6838005; doi:10.3389/fendo.2019.00720)

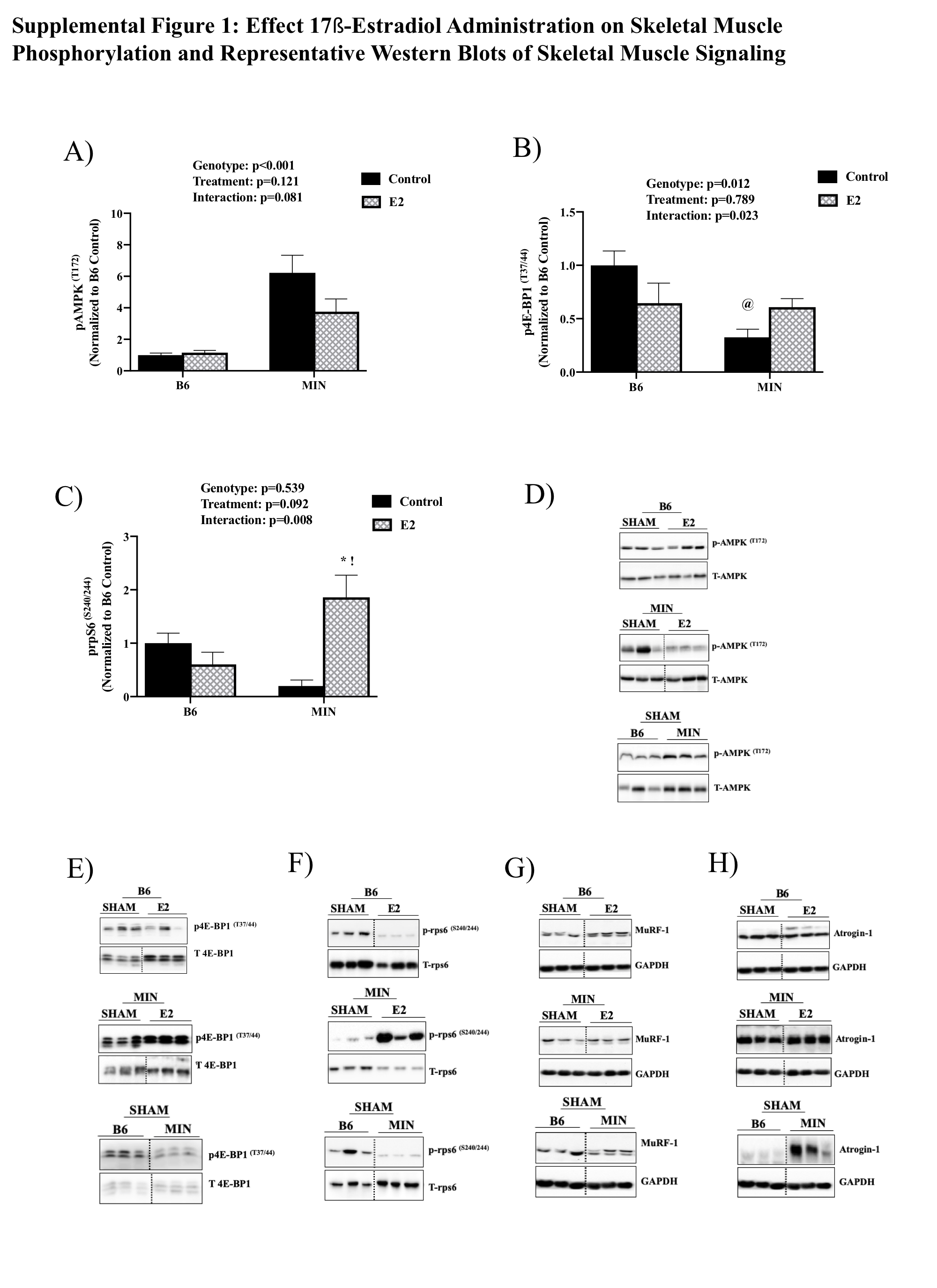

Supplement: Supplementary file 1 [file Image_1.TIFF]
